# Iliac intramedullary stabilization for Type IIIA fragility fractures of the pelvis

**DOI:** 10.1038/s41598-020-77560-7

**Published:** 2020-11-23

**Authors:** Shingo Okazaki, Masahiro Shirahama, Ryuki Hashida, Mitsuhiro Matsuura, Shiro Yoshida, Kenjiro Nakama, Hiroo Matsuse, Naoto Shiba

**Affiliations:** Department of Orthopaedic Surgery, Kurume University School of Medicine, 67 Asahi, Kurume City, Fukuoka, 830-0011 Japan

**Keywords:** Trauma, Fracture repair, Orthopaedics

## Abstract

There have been few reports on fixation of Rommens classification Type IIIA fragility fractures of the pelvis (FFPs). Here, we present our less invasive surgical technique, called iliac intramedullary stabilization (ILIS), for the internal fixation of Type IIIA FFPs. The technique involves a closed reduction, termed the femur internal rotation reduction method (FIRM), whereby the fracture fragments are repositioned using lateral rotators by internally rotating the femur while the patient is in the prone position. Two iliac screws are inserted on the ilium bilaterally via the supra-acetabular bone canal during FIRM and connected with two transverse rods and two cross connectors. We refer to this internal fixation procedure as ILIS. We retrospectively recruited patients with Type IIIA fractures, treated using this procedure, at our institute between October 2017 and October 2019. We evaluated operative and post-operative outcomes. We enrolled 10 patients (9 women and 1 man; mean age, 85.2 years) who were followed up for over 6 months. All patients suffered FFPs after falling from a standing position. The mean operative time was 145.1 (range, 94–217) minutes, and the mean blood loss was 258.5 (range, 100–684) ml. All patients were allowed full weight bearing from post-operative day 1. All patients achieved bone union and regained their pre-injury walking ability at 6 months after surgery without evident secondary displacement. In conclusion, our ILIS technique allows less invasive internal fixation of Type IIIA FFPs with adequate stability for full weight bearing from post-operative day 1.

## Introduction

Fragility fractures of the pelvis (FFPs) can result from low-energy trauma in elderly individuals with osteoporosis. The incidence of FFPs is increasing with within the current aging population as a consequence of the increasing prevalence of osteoporosis. A recent study reported that the incidence of FFPs in Finland increased from 73 per 100,000 to 364 per 100,000 from 1970 to 2013^1^. If the elderly population continues to grow at the current rate, the number of patients with FFPs is estimated to increase 2.4-fold by 2030^1^. Compston et al. reported that fractures resulting from osteoporosis become increasingly common in women aged over 55 years and men aged over 65 years^2^, and can therefore be expected to show a similar increase in coming years.

In 2013, Rommens et al. proposed a system for the classification of FFPs. In this classification, FFPs are categorised as Types I to IV. Type III FFPs represent displaced unilateral posterior lesions; Types IIIA, IIIB, and IIIC involve fractures of the iliac region, sacroiliac joint, and sacral region, respectively. Rommens classification Type III FFPs (hereafter, Type III FFPs) account for 11% of total FFPs, with Type IIIA FFPs being the most common, comprising 8.2% of all FFPs^3^.

Types IIIB and IIIC can be reduced by traction of the lower limb and the use of spinal instruments and are generally treated with minimally invasive techniques involving a transiliac internal fixator (TIFI), iliosacral (IS) screws, cement augmented IS screws, or trans-sacral bars^4–9^. By contrast, Type IIIA fractures can be treated by open reduction and internal fixation (ORIF). In addition to plate fixation with through the first window of the ilioinguinal approach, either pubic screw insertion for fixation of anterior lesion or anterior external fixation can be performed. To the best of our knowledge, there are few reports that described less invasive surgery for Type IIIA fractures^4,9–13^.

In the present report, we present our surgical technique, termed iliac intramedullary stabilization (ILIS), for the internal fixation of Type IIIA FFPs. The method for closed reduction used in this procedure is termed the femur internal rotation reduction method (FIRM).

## Methods

### Ethics approval and consent to participate

All procedures performed in this study were in accordance with the ethical standards of the institutional and/or national research committee and with the 1964 Helsinki Declaration and its later amendments or comparable ethical standards. Informed consent was obtained from all individual participants included in the study. The study was approved by the Ethical Committee of Kurume University (No. 18073). All individual participants included in the study provided informed consent for publication.

### Study population

We retrospectively recruited patients who were diagnosed with Type IIIA FFPs and who underwent ILIS at our hospital between October 2017 and October 2019. Inclusion criteria were completion of follow-up evaluations for ≥ 6 months and the availability of pre- and post-operative computed tomography (CT) data. Exclusion criteria were as follows: a follow-up period of less than 6 months and a lack of pre- and post-operative CT data. We evaluated the operative time, intraoperative blood loss, the walking ability, visual analogue scale (VAS) score, and complications. In addition, radiographic evaluation was also performed using CT data. The maximum displacements of the iliac fractures were measured before surgery, after surgery, and at 6 months after surgery.

### Surgical technique

**Figure 1:**
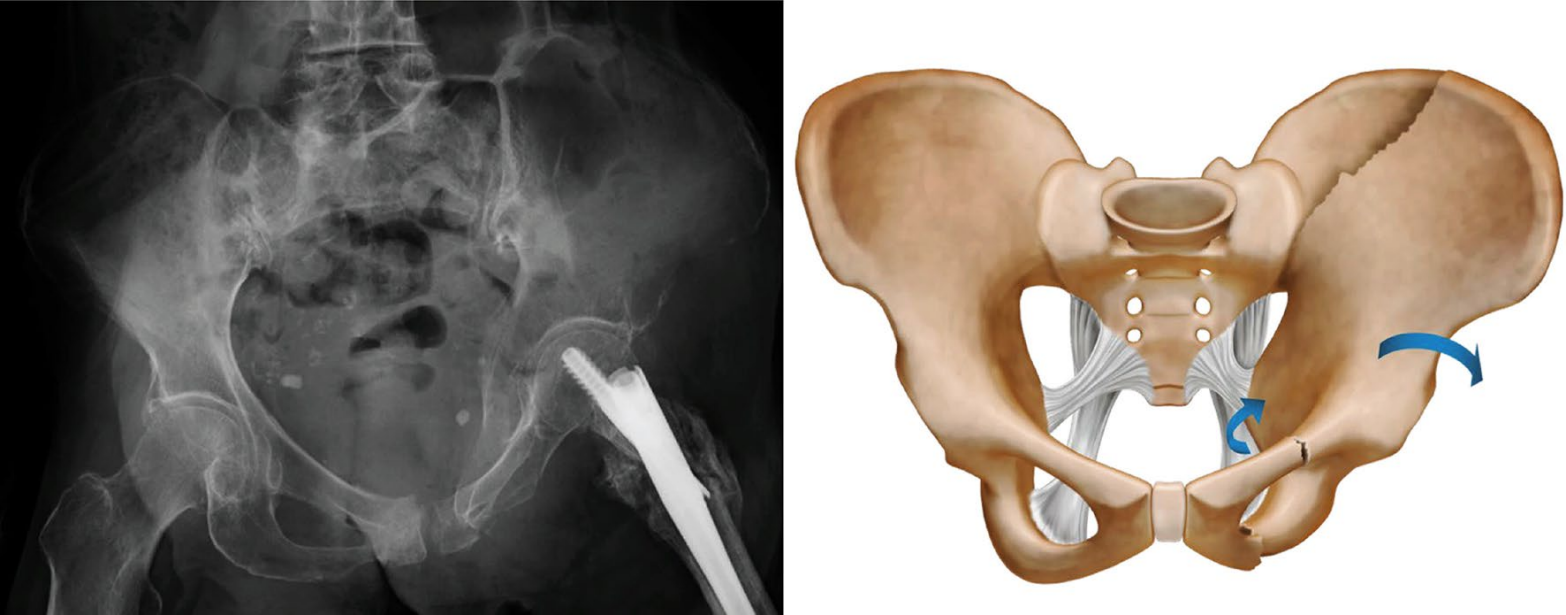
Representative plain radiographs of a Type IIIA fragility fracture of the pelvis (left). Diagram of a Type IIIA fragility fracture of the pelvis. In the case of Type IIIA fragility fractures of the pelvis, the sacrotuberous and sacrospinous ligaments are intact, the ilium is externally rotated, and the obturator foramen on the fractured side appears small (right).

**Figure 2:**
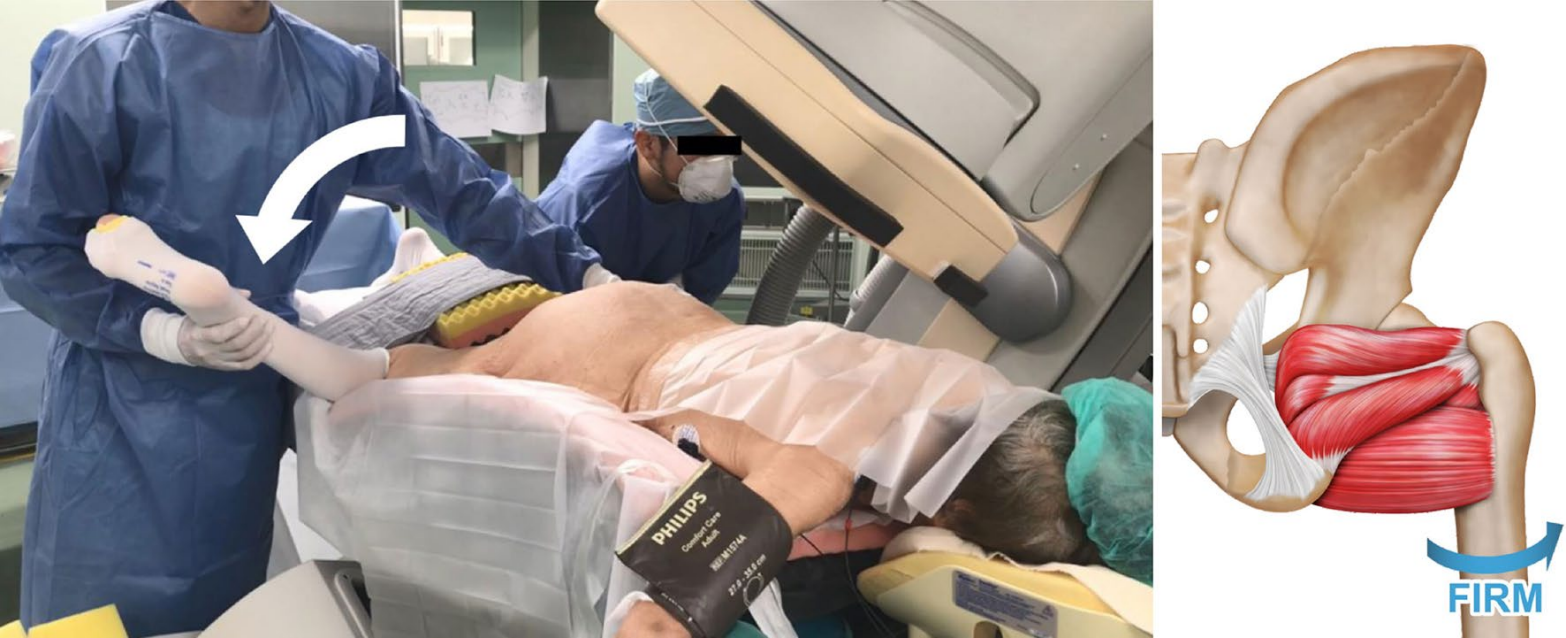
Photograph of the femur internal rotation reduction method (FIRM) used for iliac intramedullary stabilization. Reduction is performed by internal rotation of the femur with the knee bent and ankle held while the patient is in the prone position (left). During the femur internal rotation reduction method for iliac intramedullary stabilization, the femur is internally rotated to reduce the bone fragment by manipulation of the lateral rotator muscles and surrounding soft tissues (right).

In the case of Type IIIA FFPs, the sacrotuberous and sacrospinous ligaments remain intact and form anatomical borders, and fragment motion is restricted to within these borders^3,11^. The ilium is externally rotated, and the obturator foramen on the fractured side appears small on the anteroposterior (AP) view on radiographic examinations (Fig. 1). Lateral rotators are attached to the femur from the ischial spine and ischial tuberosity. The FIRM technique involves internal rotation of the femur to reduce the bone fragmentation by manipulating the lateral rotator muscles and surrounding soft tissues. Reduction is performed by holding the ankle and bending the knee with the patient in the prone position to internally rotate the femur (Fig. 2). In the AP view on fluoroscopy, the shape of the obturator foramen on the fractured side is gradually reduced. The teepee view also improves gradually. The teepee view is similar to the obturator oblique view and represents the supra-acetabular bone canal running the line from the anterior inferior iliac spine (AIIS) to the posterior superior iliac spine (PSIS) and posterior inferior iliac spine^14^. This technique aligns the supra-acetabular bone canal between the PSIS and AIIS in a relatively straight line, enabling screw insertion. Thus, reduction by FIRM and fixation by spinal instruments is achieved; we call this procedure ILIS.

**Figure 3:**
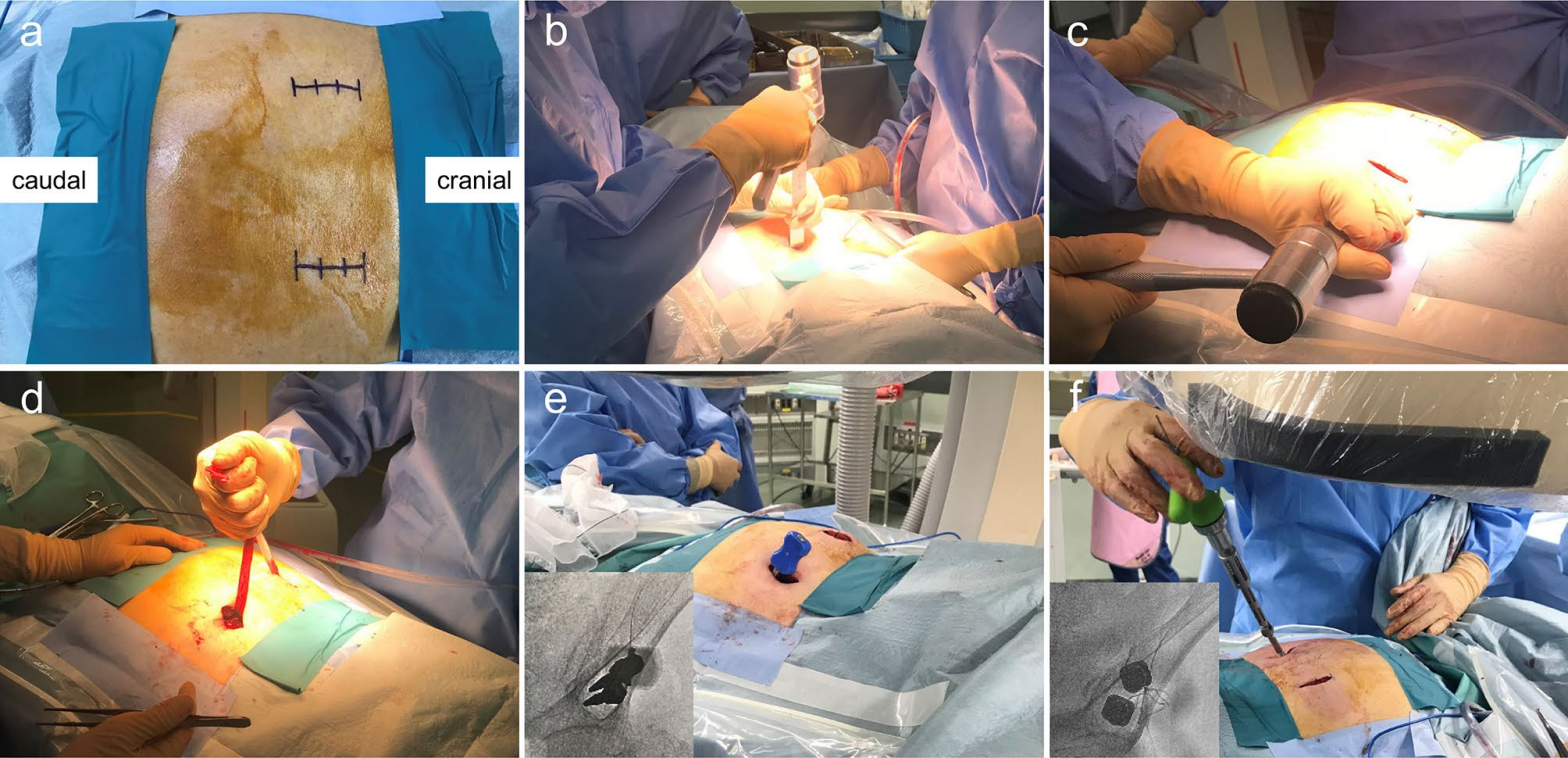
Bilateral 3–4-cm incisions are made into the skin, directly above the posterior superior iliac spine (PSIS) (**a**). An approximately 3-cm wide bone groove is created at the PSIS, to the depth of the dorsal surface of the sacrum (**b**). The spinal processes of the sacrum are cut with a chisel (**c**). A tunnel connecting the left and right sides (**d**). Two probes are inserted from the bone groove of the PSIS to the anterior inferior iliac spine (AIIS) via the supra-acetabular bone canal during the femur internal rotation reduction method (**e**). Two Expedium φ9.0 mm SAI screws are inserted into the AIIS via the supra-acetabular bone canal during the femur internal rotation reduction method (**f**).

**Figure 4:**
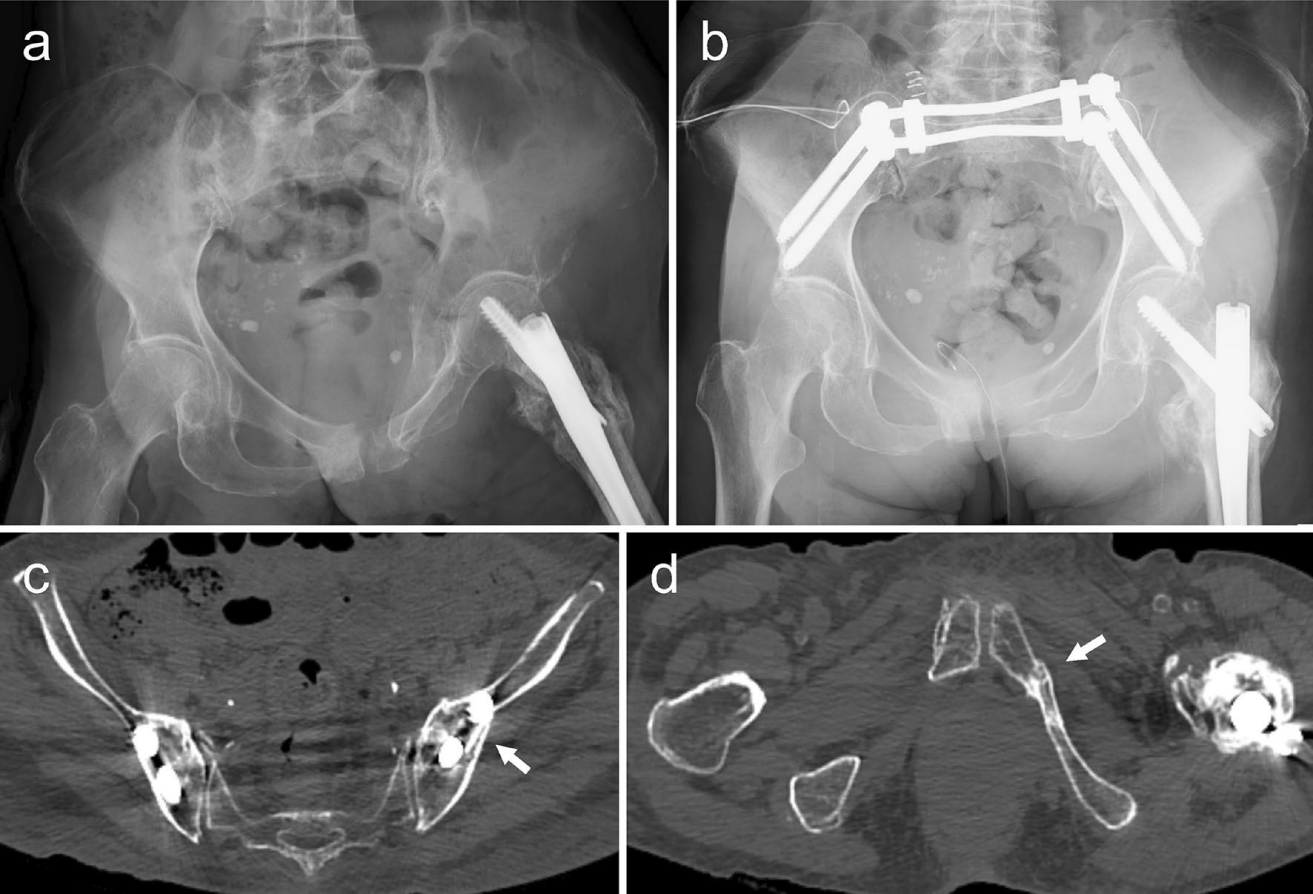
Representative plain radiographs and post-operative computed tomography images. A plain radiograph obtained after the injury (**a**). A plain radiograph obtained after treatment shows that reduction has been achieved. The obturator foramen appears almost symmetrical (**b**). Bone union of the left iliac bone (**c**). Bone union of the inferior ramus of the left pubis (**d**).

The details of the protocol as performed in our department are as follows: First, the patient is placed in the prone position, and 3–4-cm incisions are made to the skin bilaterally, directly above the PSIS to expose the PSIS subperiosteally. Next, an approximately 3-cm wide bone groove is created by resecting the bone with a chisel at the PSIS, to the depth of the dorsal surface of the sacrum. This bone groove is created to reduce implant irritation and skin complications after surgery by burying the screw heads in the PSIS. The muscles on the dorsal surface of the sacrum are dissected, and the spinal processes of the sacrum are cut with a chisel to create a tunnel connecting the left and right sides. FIRM is then performed, and two probes are inserted from the bone groove of the PSIS to the AIIS via the supra-acetabular bone canal. Guide pins are inserted, the probes are extracted, and two Expedium φ9.0 mm SAI screws (DePuy Synthes Co., Zuchwil, Switzerland) are inserted into the AIIS via the supra-acetabular bone canal (Fig. 3). Further, two screws are inserted on the contralateral side. Normally, the screws are inserted at a length of around 80–100 mm. The screw heads are located at the depth of the dorsal surface of the sacrum. Two rods are inserted through the tunnel, fastened to the screw head, and then further fastened with two cross connectors (Fig. 4a, b). If the rods collide with the dorsal surface of the sacrum and are difficult to fasten, they are bent into a gull-wing shape. Soft tissues including the thoracolumbar fascia are sutured to cover the implants at the time of closure.

## Results

We retrospectively recruited 11 patients for the present study. Of these, ten (nine women and one man) met the inclusion criteria; one patient died of heart failure before completing the required follow-up protocol. The mean follow-up period was 11.2 (range, 6–19) months. The mean age was 85.2 (range, 74–95) years, and the cause of injury was a fall from a standing position in all cases. The mean period from the time of trauma to surgery was 3.8 (range, 2–8) days, and the mean time from admission at our hospital to surgery was 2.2 (range, 1–5) days. All patients were operated upon by the first author. The mean operative time was 145.1 min (range, 94–217 min) and mean intraoperative blood loss was 258.5 ml (range, 100–684 ml). All patients were allowed full weight bearing on the operated limb from post-operative day 1 and started walking training when wound pain receded. Patients were discharged at an average of 20.9 (range, 8–35) days after surgery and transferred to a rehabilitation hospital. The walking ability at the time of discharge from our hospital was categorised as a household ambulator with a walker in six patients and a non-functional ambulator in four patients with dementia. The maximum displacements of the iliac fractures were measured using CT data before surgery, after surgery, and at 6 months after surgery. The mean displacements were 9.64 mm (range, 3.2–15.57 mm), 5.63 mm (range, 1.65–10.39 mm), and 6.07 mm (range, 1.97–11.5 mm), respectively. The mean difference in the displacement after surgery and 6 months after surgery was 0.44 mm (range, 0.04–1.52 mm). Bone union was observed by 6 months in all patients (Fig. 4c,d), and all patients also regained their pre-injury walking ability (Table 1). VAS scores at final follow-up were 0 in five patients; VAS scores could not be obtained for the other five patients because of dementia.

**Table 1:** Clinical data of participants.

Patient number	Age (years)	Sex	Pre-injury walking ability	Walking ability at the time of discharge our hospital	Walking ability at 6 months after surgery	Dementia	VAS score at the final follow-up
1	82	F	Household ambulator with walker	Non-functional ambulator	Household ambulator with walker	+	NA
2	82	F	Independent community ambulator	Household ambulator with walker	Independent community ambulator	−	0
3	95	F	Household ambulator with walker	Non-functional ambulator	Household ambulator with walker	+	NA
4	86	F	Independent community ambulator	Household ambulator with walker	Independent community ambulator	−	0
5	74	F	Community ambulator with cane	Household ambulator with walker	Community ambulator with cane	−	0
6	87	F	Household ambulator with walker	Household ambulator with walker	Household ambulator with walker	−	0
7	91	F	Non-functional ambulator	Non-functional ambulator	Non-functional ambulator	+	NA
8	80	M	Independent community ambulator	Household ambulator with walker	Independent community ambulator	−	0
9	88	F	Household ambulator with walker	Household ambulator with walker	Household ambulator with walker	+	NA
10	87	F	Non-functional ambulator	Non-functional ambulator	Non-functional ambulator	+	NA

VAS, visual analogue scale; NA, not available.

In terms of post-operative complications, no cases of evident secondary displacement or implant rupture were observed. However, delayed wound healing requiring bedside secondary sutures occurred in two patients, one of whom developed a pressure ulcer on the opposite side of the wound requiring secondary sutures at 8 months after surgery. The pressure ulcer was treated with bedside lavage and negative pressure wound therapy. Deep infection occurred in one patient at 3 months after surgery, and it was treated with implant removal and lavage. This patient did not show any change in activities of daily living after implant removal and lavage, because bone union was observed at the time. After treatment, there were no obvious problems in the clinical course.

## Discussion

According to Rommens et al., surgery is the appropriate therapy for Type III FFPs, given that these fractures are difficult to treat with conservative therapy alone as they are unstable. The authors also highlight that less invasive fixation is desirable for FFPs. The goal of Type IIIA FFP is recovery of function and stability, which is considered more important than achieving complete anatomical reduction^4,15^.

The procedure described in this study, ILIS, is a closed reduction and internal fixation (CRIF) technique for Type IIIA FFPs. While complete anatomical reduction is difficult through the FIRM alone, the supra-acetabular bone canal can be reduced relatively linearly, allowing implant insertion. Two intramedullary φ9.0-mm screws, inserted into the ilium, provide a similar reduction to intramedullary nails for diaphyseal fractures of the limbs and provide internal fixation. Insertion of two intramedullary screws also reduces the possibility of post-operative rotation and displacement of bone fragments, which can occur when a single screw is used. Achieving sufficient fixation with screws inserted only into the fractured side is considered difficult because of the osteoporotic bone and because the screws used in this technique have partial threads. The fixation method presented here can achieve sufficient fixation through the insertion of two screws as well as anchoring screws on the contralateral side. Although we did not perform any fixation of the anterior pelvis, there was no evidence of secondary displacement in our series. We believe that anterior fixation is not necessary in this procedure.

There are several techniques for pelvic fractures, including the use of LCII and IS screws, that can be performed in the supine position and percutaneously^7,10,16^; our technique must be performed in the prone position and requires 3–4-cm skin incisions. Although our technique seems more invasive than the percutaneous techniques, it could repair Type IIIA FFPs less invasively and with adequate stability for full weight bearing from post-operative day 1 in our series.

Type IIIA fractures are similar to lateral compression Type II (LCII) fractures^3,17^. A previous report about the treatment of LCII fractures using an ilioinguinal approach shows that mean operative time and mean intraoperative blood loss were 159.7 min and 563.6 ml, respectively, in the isolated posterior fixation group and 211.1 min and 875.5 ml, respectively, in the anterior and posterior fixation group^18^. In our series, the mean operative time was 145.1 min and the mean intraoperative blood loss was 258.5 ml. Our technique produced less intraoperative blood loss than conventional ORIF. This may be due to the use of a CRIF technique, the need for small skin incisions (3–4 cm bilaterally), and less muscle dissection than conventional ORIF. Although there were some patients who were taking anticoagulant and antiplatelet medications due to their comorbidities, we performed surgery at an average of 2.2 days from admission at our hospital. This may explain why we observed a maximum blood loss of up to 684 ml.

**Figure 5:**
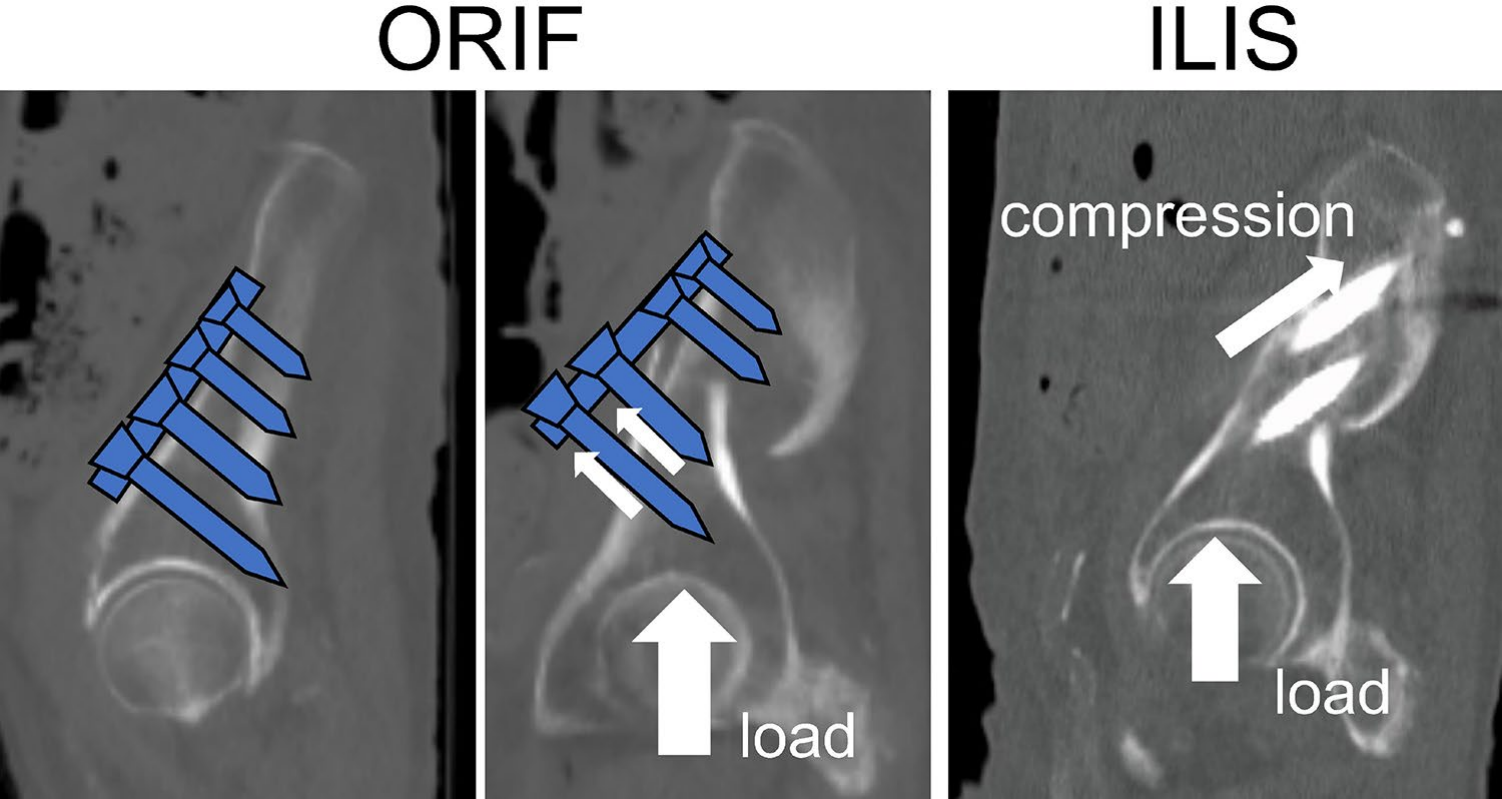
Illustration demonstrating the approaches of open reduction and internal fixation as well as iliac intramedullary stabilization. In open reduction and internal fixation, the direction of the force exerted by early weight bearing can dislodge the screws. In iliac intramedullary stabilization, screws are occupied in the supra-acetabular bone canal so that the load compresses the fracture site, allowing early weight bearing.

A previous report also demonstrated that the mean time to weight bearing was 3 weeks^18^. This appears to be a rapid recovery of the weight bearing ability, because significant weight bearing is usually possible by 6 weeks post-operatively in the case of pelvic lateral compression fractures^19^. In contrast, we allowed full weight bearing from post-operative day 1, and the secondary displacement was very small (average 0.44 mm). With respect to the force applied to the implant during weight bearing, in ORIF, loading generates force in a direction that can dislodge the screws. Thus, early weight bearing is difficult after ORIF. With the present method, iliac intramedullary screws are occupied in the supra-acetabular bone canal like intramedullary nails; therefore, the load compresses the fracture site, enabling early weight bearing (Fig. 5). Furthermore, full, early, post-operative weight bearing was thought to be possible because the ligaments remain intact in FFPs.

Rommens et al. reported that fracture progression can occur in FFPs. They described a case of one patient who had an additional contralateral fracture after operative treatment of the fractured side^20^. Our technique involves the insertion of two screws into the ilium bilaterally and can be consider ILIS, which can prevent fracture progression in Type IIIA FFPs. Moreover, there were no cases of fracture progression in our series.

This study has several limitations that should be acknowledged. First, the mean follow-up period was only an average of 11.2 months after surgery. Future studies involving longer periods of follow-up are needed to confirm the long-term outcomes. Second, the sample size was very small. Due to the novelty of the technique, it was difficult to conduct a multi-institutional study and to perform surgery for a sufficient number of Type IIIA FFPs in a single institution. It is unclear whether our approach would be suitable for all Type IIIA FFPs. Further investigations with an increased number of patients are required to clarify this aspect. Third, to the best of our knowledge, there are few articles on the treatment of Type IIIA FFPs. Therefore, we could not sufficiently compare ILIS with conventional ORIF for Type IIIA FFPs. While we compared ILIS with ORIF for LCII fractures, LCII fractures are caused by high energy trauma and are difficult to compare with Type IIIA FFPs. Because of these limitations and the inherent risk of bias due to the retrospective design, we cannot generalize the results or conclusions to other populations or indications.

In conclusion, we treated Type IIIA FFPs with our novel ILIS technique, which is facilitated by the FIRM for easy screw insertion. In our series, the ILIS technique allowed less invasive internal fixation of Type IIIA FFPs with adequate stability for full weight bearing from post-operative day 1. As a result, early walking training was possible, and patients regained their pre-injury walking ability. Our study suggests that this method represents an effective operative approach for the treatment of Type IIIA FFPs to preserve patients’ walking ability.

## Data Availability

The datasets generated and analyzed during the current study are available from the corresponding author on reasonable request.
